# In depth profiling of dihydrolipoamide dehydrogenase deficiency in primary patients fibroblasts reveals metabolic reprogramming secondary to mitochondrial dysfunction

**DOI:** 10.1016/j.ymgmr.2024.101172

**Published:** 2024-12-16

**Authors:** Uri Sprecher, Jeevitha Dsouza, Monzer Marisat, Dinorah Barasch, Kumudesh Mishra, Or Kakhlon, Joshua Manor, Yair Anikster, Miguel Weil

**Affiliations:** a The Shmunis School of Biomedicine and Cancer Research, The George S. Wise Faculty for Life Sciences, Sagol School of Neurosciences, Tel Aviv University, 6997801 Tel Aviv, Israel; b Mass Spectrometry Unit, Institute for Drug Research, School of Pharmacy, Hebrew University of Jerusalem, Jerusalem 9112102, Israel; c Department of Neurology, The Agnes Ginges Center for Human Neurogenetics, Hadassah Hebrew University Medical Center, Jerusalem 9112001, Israel; d Faculty of Medicine, Hebrew University of Jerusalem, Ein Kerem, Jerusalem 9112102, Israel; e Edmond and Lily Safra Children's Hospital, Sheba Medical Center Tel-Hashomer, 52621, Israel; f School of Medicine, Faculty of Medicine and Health Science, Tel Aviv University, 6997801 Tel Aviv, Israel

**Keywords:** Dihydrolipoamide dehydrogenase deficiency, Metabolic disorder, Patient study, Primary skin fibroblasts, Mitochondrial activity, Image based high content analysis, Metabolomics, Next generation sequencing, RNA -seq

## Abstract

Dihydrolipoamide dehydrogenase (DLD) deficiency is an autosomal recessive disorder characterized by a functional disruption in several critical mitochondrial enzyme complexes, including pyruvate dehydrogenase and α-ketoglutarate dehydrogenase. Despite DLD's pivotal role in cellular energy metabolism, detailed molecular and metabolic consequences of DLD deficiency (DLDD) remain poorly understood. This study represents the first in-depth multi-omics analysis, specifically metabolomic and transcriptomic, of fibroblasts derived from a DLD-deficient patient compound heterozygous for a common Ashkenazi Jewish variant (c.685G > T) and a novel North African variant (c.158G > A). The investigation reveals significant metabolic disruptions that distinguish the cellular phenotype of DLDD from other metabolic disorders and healthy controls. Employing a range of cellular and molecular techniques, including live-cell imaging, mitochondrial activity assays, immunofluorescence, transcriptomics and metabolomic analysis, we compared DLDD fibroblasts with fibroblasts from glycogen storage disease type 1 A (GSD1a) patients and healthy controls (HC) subjects. Our metabolomics analysis identified significant alterations in mitochondrial metabolism, particularly reduced glycine cleavage, altered one carbon metabolism and serine catabolism. Transcriptome profiling highlighted dysregulation in genes associated with metabolic stress and mitochondrial dysfunction. Our findings highlight reduced mitochondrial activity and respiratory capacity in DLDD fibroblasts, similar to observations in GSD1a fibroblasts. This multi-omics approach not only advances our understanding of the pathophysiology of DLDD, but also illustrates the potential for developing targeted diagnostics and therapeutic strategies.

## 1 Introduction

Dihydrolipoamide dehydrogenase (DLD) is a flavoenzyme essential for the activity of cellular dehydrogenase complexes, namely pyruvate dehydrogenase, α-ketoglutarate dehydrogenase, branched-chain α-ketoacid dehydrogenase, 2-oxoadipate dehydrogenase (generating glutaryl CoA *en route* to acetyl CoA in the lysine degradation pathway), and the glycine cleavage system [ [1], [2], [3]].

DLD deficiency (DLDD) results in a rare autosomal recessive condition, with ∼30 cases reported worldwide (Inborn Metabolic Diseases book by Saudubray et al. 7th edition, chapter 11), together with a recent natural history study that analyzed a population of another 53 patients [4].

Notably, a high carrier prevalence is detected within the Ashkenazi-Jewish population and Bedouin clan in Southern Israel, resulting in a significantly higher incidence of DLDD in these groups (Dr. Staretz-Chacham, communicated at the 2023 SSIEM annual symposium). The clinical manifestations of DLDD are notoriously diverse and fall into three predominant phenotypes: The classic form manifests in early childhood with signs including progressive hypotonia, growth failure, hypoglycemia, and acute metabolic acidosis episodes. The neonatal onset form can lead to early fatality, while survivors might experience profound neurological issues, including intellectual disability and seizures. Lastly, another manifestation involves liver failure, and presents as acute nausea, vomiting, and liver failure, which may deteriorate to hepatic encephalopathy and hypocoagulation. This form appears anytime from the neonatal period to late adulthood [[1], [2], [3]]. A particularly uncommon presentation of DLDD in all its forms is riboflavin-responsive muscle weakness, characterized by elevated lactate levels, and, in some instances, myoglobinuria. Additionally, several individuals remain asymptomatic for life. The Ashkenazi-Jewish common founder mutation is mostly linked to the liver phenotype [1].

Here we present for the first time an in-depth multi-omics analysis of fibroblasts from a DLDD compound heterozygous patient with the common Ashkenazi-Jewish variant (c.685G > T) and a novel North African variant (c.158G > A) associated with a more severe phenotype. The patient manifests a significant liver disease. Cell-based analysis of fibroblasts from this patient demonstrated changes in the metabolomic profile and perturbations in mitochondrial activity and metabolism, specifically reduction in glycine cleavage. We also compared, using patients' primary skin fibroblasts, the profiles of DLDD and another hepatic metabolic disorder common in the Ashkenazi population, GSD1a. We show that these two disorders cluster together and are separated from control cells, suggesting a common metabolomic footprint. This novel personalized cell based multi-omics analysis of a patient with a rare genetic disorder adds potential valuable information on the disease phenotype. The affected metabolic pathways discovered might be targeted for clinical assessment and treatment.

## 2 Materials and methods

**Chemicals and reagents were purchased from Merck-Sigma-Aldrich or as otherwise stated.**

**Cell culture.** Primary Skin fibroblasts were isolated from skin biopsies of one DLDD patient and two GSD1a patients (See Table 1 for sample information). Informed written consent to participate in the study was obtained from all individuals or their parents in the case of minors. The study was approved by the ethics committee of the Institutional Review Board of the Sheba Medical Center (5722–18-SMS). Age and gender matched healthy controls were purchased from the Coriell Institute (Camden, NJ, USA) (see Table 1). Primary fibroblasts were expanded in DMEM supplemented with 1 % MEM Sodium Pyruvate, 1 % penicillin-streptomycin-amphotericin (PSA, Biological Industries, Beit HaEmek, Israel), 10 % heat inactivated FBS, and 1 % 100× non-essential amino acids solution (NEAA) in polystyrene plastic 75-cm^2^ culture flasks (Corning, NY, USA) at 37 °C with 5 % CO_2_. Cell passages and expansion of human skin fibroblasts were performed at 80–100 % confluency using 0.25 % Trypsin-EDTA (Biological Industries) for 2 min for cell detachment, followed by addition of twice the volume of a complete culture media to neutralize the enzyme. Cells were subsequently centrifuged at 1200 rpm (244 RCF) for 5 min before their pellets were resuspended in 1 ml full medium for cell counting using TC10 automated cell counter (BioRad, Hercules, CA, USA) before re-plating. All experiments were performed using skin fibroblasts at passages 8–16.

**Table 1:** - Cells.

Cell Type	Cell id	Age	Gender	Disease mutation	sample paired to the patient sample
GSD1A	361	15	Female	c.247C > T (p.Arg83Cys); c.247C > T (p.Arg83Cys)	
GSD1A	75,858	19	Male	c.247C > T (p.Arg83Cys); c.247C > T (p.Arg83Cys)	
DLDD	53,273	7	Male	c.685G > T (p.Gly229Cys); c.158G > A (p.Gly53Glu)	
HC	GM00038	9	Female		361
HC	GM17507	16	Male		75,858
HC	GM00498	3	Male		53,273

**Live Imaging.** DLDD, GSD1a and HC skin fibroblasts were seeded at 1400 cells per well and cultured in microscopy-grade 96-well plates (Cellvis P96–1.5H-N). Following 24 h in complete medium (DMEM with glucose,10 % FBS, PSA, NEAA and sodium pyruvate), cells were washed to remove traces of serum and starvation medium (DMEM serum free, glucose and pyruvate free, with PSA) was applied for 24 and 48 h, or for 48 h followed by 24 h with complete glucose-containing medium (72 h). Before imaging, the cells at the end of their respective time points were washed and a mix of live fluorescent dyes in HBSS was added to each well for 20 min at 37 °C in a 5 % CO_2_ incubator. The final concentrations of the different stains were: 1.6 μM Hoechst 33342, 0.05 mM TMRE and 0.4 mM calcein-AM Green (Thermo-Fisher Scientific). Following this incubation, cells were washed with HBSS, and plates were transferred to an Operetta G1 system for image acquisition at 20× magnification under environmental control conditions at 37 °C and 5 % CO_2_. All the assay parameters (including the acquisition exposure times, objective, and the analysis parameters) were kept constant for all assay repetitions. Images were analyzed by a customized image analysis protocol designed for the experiment using Harmony image analysis software. Signal Outlier value detection was used to remove outlier wells using the Prism Route method (Q = 10 %).

**Mitochondrial activity assays.** Mitochondrial bioenergetics (OCR and ECAR) of primary skin fibroblasts was quantified in real-time using the Seahorse extracellular XFe96 flux analyzer (Agilent, Santa Clara, CA, USA). Cells were plated in XFe96 cell culture plates at a density of (40,000 cells/well). Cells were then washed in assay media (XF Base media (Agilent) with glucose (10 mM), sodium pyruvate (1 mM) and l-glutamine (2 mM) (Gibco, Waltham, MA, USA), pH 7.4 at 37 °C). OCR and ECAR were measured using Seahorse's Mito Stress assay (Agilent), with the preset automatic addition of oligomycin (1.5 μM), carbonyl cyanide 4-(trifluoromethoxy) phenylhydrazone (FCCP; 2 μM) and antimycin A and rotenone (0.5 μM each)).

**Immunofluorescence.** 1800 cells per well were plated in 96 well plates (Cellvis P96–1.5H-N), cultured overnight for attachment. Medium treatment was then applied for 72 h- 48 h in starvation medium, followed by a complete medium replacement for 24 h as described above. Cells were fixed in 4 % paraformaldehyde in PBS, washed three times in PBS, permeabilized in 0.1 % Triton X-100 in PBS for 5 min and blocked-in blocking buffer (5 % FBS & 2 % BSA in PBS) for 1 h. Cells were then incubated with primary antibodies (see Table 2) overnight In 4 °C, followed by three washes in 0.05 % Triton X-100 in PBS and incubation with secondary antibodies and counterstaining with 1.6 mM Hoechst 33342 (1:1000) and 0.02 mM phalloidin Atto 565 (1:400) for 1 h. Cells were subsequently washed three times in PBS. Images were acquired with 20× magnification using the Operetta High content imaging system. The experiment was repeated 3 times per primary antibody, where the assay parameters (including the acquisition exposure times, objective, and the analysis parameters) were kept constant for each antibody. Images were analyzed by the Harmony image analysis software using a customized image analysis protocol and quantified for intensity levels of each antibody. Signal Outlier value detection was used to remove outlier wells using the Prism Route method (Q = 10 %).

**Table 2-:** Antibodies.

Antibody	Company	Dilution	Method
DRP1	Merck	1:100	IF
TIMM13	Proteintech	1:100	IF
COX 17	Proteintech	1:100	IF
ALR	Proteintech	1:50	IF
Donkey anti-rabbit Alexa Fluor 488 conjugated	BETHYL	1:1000	IF
Donkey anti-goat Alexa Fluor 488 conjugated	Thermo	1:1000	IF

**Metabolomics.** Metabolomics sample preparation was performed on one DLDD, two GSD1a, and three HC fibroblast lines. At the end of the 72 h culture condition as described above, cells were washed with PBS to remove the culture media. To extract metabolites cells were then washed twice with cold saline (0.9 % NaCl solution) and detached using a cell scraper. The samples were centrifuged at 1200 rpm for 3 min at 4 °C. The supernatant was removed and ice cold 90 % methanol in H_2_O was added to each cell pellet. Pellets were sonicated in three cycles of 3 min each at 4 °C, snap frozen in liquid nitrogen for 3 min, and thawed. The samples were then centrifuged at 18,000 rpm for 5 min at 4 °C. The supernatants were transferred to new tubes and stored in liquid nitrogen until analysis. To capture a broad spectrum of metabolites, we used the AbsoluteIDQ® p180 kit (Biocrates Life Sciences AG, Innsbruck, Austria), targeting 40 AC, 42 AA/biogenic amines, 90 phospholipids, 15 sphingolipids, and hexose, following the manufacturer's instructions. Briefly, 10 μL of calibration standards, quality controls, and samples were added to the respective wells of the 96-well-based Biocrates sample preparation plate containing a mix of internal standards. After drying the samples under nitrogen, 50 μL of 5 % phenyl-isothiocyanate solution were added to each well for derivatization. After incubation for 25 min and subsequent evaporation to dryness under nitrogen, 300 μL of 5 mM ammonium acetate in methanol were added for metabolite extraction, stirred for 30 min and centrifuged. The extracts were diluted with 250 μl of 40 % methanol/water. The extracts were analyzed by liquid chromatography with tandem mass spectrometry (LC-MS/MS). This system comprised the Nexera UHPLC system (Shimadzu, Kyoto, Japan) coupled to a Triple Quad™ 5500 mass spectrometer (Sciex, Framingham, MA, United States) in electrospray ionization (ESI) mode. AA and biogenic amines were analyzed *via* LC-MS in a positive mode. 5 μL of the sample extract were injected to Biocrates AbsoluteIDQ® p180 kit UHPLC column, 2.1 × 50 mm, protected by a VanGuard® pre-column (Waters, Milford, MA, United States) at 50 °C using a 5.8 min solvent gradient employing 0.2 % formic acid in water and 0.2 % formic acid in acetonitrile. 20 μl of the sample extracts were used in the flow injection analysis (FIA) in the positive mode to capture AC, glycerophospholipids, sphingolipids and hexoses. All FIA injections were carried out using the Biocrates FIA Solvent. All metabolites were identified and quantified using isotopically labeled internal standards and multiple reaction monitoring (MRM). Metabolomics analysis was performed by Metaboanalyst 6.0.

**RNA-seq**. RNA-seq library preparation was performed on one DLDD fibroblast, three HC fibroblasts and two GSD1a fibroblasts. Cells were cultured for 72 h as described above and then washed in PBS to remove traces of culture media and were kept frozen in freezing medium (FBS with 10 % DMSO) until use. After thawing, RNA extraction on cell pellets was performed using Qiagen's RNeasy kit according to manufacturer's instructions. RNA was evaluated by TapeStation 4200 (Agilent). RNA-Seq libraries were prepared with the TruSeq® Stranded mRNA Library Prep kit. RNA libraries were evaluated by the Qubit3.0 fluorometer (Invitrogen) and TapeStation 4200 (Agilent). Sequencing was done with a NextSeq 500/550 High Output Kit v2.5 (75 Cycles) on a NextSeq500 sequencer, all according to manufacturer's instructions (Illumina). Sequencing was performed at the Genomics Research Unit, supported by The Alfredo Federico Strauss Center for Computational Neuro-imaging, faculty of Life Sciences, 10.13039/501100004375Tel Aviv University. Fastq files were generated and quality control of files was performed using fastqc (0.12) and multiqc softwares [5,6]. Illumina adaptor sequences were trimmed using Trimmomatic (0.39) [7] followed by another fastqc and multiqc report. Sample replicates were merged and sequencing reads were then aligned to the GRCh38 human genome using hisat2 [8,9]. Output files were sorted, indexed and used for calculation of mapping statistics using SamTools (1.18) [10]. Reads mapping to mitochondrial DNA were removed with SAMtools. PCR-duplicates were removed using Picard MarkDuplicated (4.0.1.1) [11]. Reads were then counted using Htseq [12]. Further processing of the data, QC, normalization and differential expression analysis were performed using the R package DeSeq2 [13]. Likelihood Ratio Test (LRT) was performed using DESEQ2 and cluster analysis was performed using the R package DegReport [14]. We implemented this methodology to expand the analysis of differential gene expression, taking into consideration the statistical limitations due to the reduced sample numbers (1 DLDD and 2 GSD1a), The aim of the LRT was to identify genes that exhibit significant differences in expression across all tested groups (*i.e.* DLDD, GSD1a, HC-DLDD, HC-GSD1a). Conceptually, it was used to test for differences across multiple groups in a similar way as ANOVA. Using the LRT test we compared the full model to the reduced model with just the intercept which assumes no effect of any variable on gene expression. The LRT tests whether including any of the group variables in the model explains a significant portion of the variation in gene expression. The result outputs *p* values which are computed based on deviance between the full and reduced model, and not Log 2-fold change(L2FC), which serves as an advantage for situations with a low sample size. We then follow a set of genes that show overall differences in expression patterns between all groups and searched for significant genes with a pattern that is consistent with both disease groups (*i.e.*, DLDD and GSD1a). This was achieved using DEGreports that applies gene expression changes to group together different genes. We applied degPatterns tool using hierarchical clustering based on pairwise correlations and then cutting the hierarchical tree to find group of genes with similar expression profiles (Fig. 2C, left). This tool cuts the tree to force larger inter-cluster variability than intra-cluster variability. Enrichment analysis (Fig. 2C, right) was performed using clusterprofiler based on comparison to the GO database [15,16]. Other packages used for analysis and visualization are PCAtools [17],ggplot2 [18] and Plotly [19].

**Data Analysis & Statistics.**

Generally, all data processing stages in all experiments were performed using Python 3 and R. Prime Image analysis was customized for each experiment using the Harmony 4.8 image-analysis software. Image & Data analysis was inspired by Caicedo et al [20] Generally, *p*-values were generated using statistical testing by 2 tailed unpaired *t*-tests which were performed to compare groups using GraphPad Prism 9, unless otherwise mentioned. For Seahorse, immunofluorescence and Live imaging N equals the number of wells for each group. For multi-omics experiments N equals number of samples analyzed. Throughout the manuscript, *p* < 0.1 was considered significant as supported by these previously published works [[21], [22], [23]]. As extremely small sample sizes such as shown in this manuscript result in lower statistical power, a higher *p*-value threshold allowed us to identify potentially meaningful differences between groups. Moreover, regarding the data presented in this manuscript, we believe that even a small effect size may have a meaningful biological implication. The higher threshold used here allowed us to identify these small effect sizes and to generate candidates for validation in larger studies that can be performed in the future based on this study. We are aware of the limitations of our study as the extremely small sample size used might be insufficient to produce the biological variability needed for drawing precise conclusions. However, using conventional *p* value thresholds (*i.e.*, 0.05) would have lowered our chances to detect important trends in the data. Since our sample size was so small, we chose to focus on effect sizes, to use a higher p value threshold and to implement various advanced methodologies that allowed us to find important biological patterns that could now be further investigated in more comprehensive studies in the future.

For Scripts used see - https://github.com/Urisprecher.

## 3 Results

*Case presentation of a DLDD patient.*

The patient is an 11-year-old male who first came to medical attention at the age of 12 h with severe lactic acidosis, decreased *per os* intake and lethargy. The diagnostic workup included molecular testing, which identified compound heterozygosity for c.158G > A (p.Gly53Glu) and c.685G > T (p.Gly229Cys), confirming a diagnosis of DLDD. The patient has experienced recurrent metabolic crises with decreased consciousness and vomiting, each necessitating admission at the intensive care unit, several times a year, for the first 6 years of life. The crises, mostly triggered by viral illnesses, were characterized at presentation by acidosis of pH 7.183–7.303 (norm: 7.32–7.43) with elevated lactic acid to 41–163 mg/dL (normal range: 6–18 mg/dL), hypoglycemia in the range of 50–60 mg/dL (normal range: 70–110 mg/dL), hyperammonemia to 109–359 μg/dL (normal range: 33–102 μg/dL), and progressive hypotonia. Laboratory findings also included elevation of liver enzyme levels in the blood, with both aspartate transaminase (AST) and alanine transaminase (ALT) peaking up to 5000 IU/L (upper limit of normal range is 60 IU/L for AST and 45 for ALT IU/L), alkaline phosphatase peaking at 798 IU/L (normal range: 145–320 IU/L), and a moderate elevation in γ-glutamyl transferase (GGT) to 118 IU/L (normal range: 10–49 IU/L). Crises were treated with dextrose-containing intravenous fluids (5 % dextrose) at 150 % of maintenance, and close monitoring. After the 6th year of life, a significant reduction in crisis frequency was noted to one every 12–24 months.

The patient exhibits developmental delays. He began to walk at 22 months, and is unable to compose complex sentences as expected from children his age. Neurocognitive evaluation found him to have limited insight and mild cognitive impairment, although a formal IQ testing has not been performed. He was diagnosed with stereotypy, hyperactivity and attention deficiency for which he takes risperidone 0.5 mg/day. He attends special education classes, and receives physiotherapy, speech therapy, and hydrotherapy.

In our clinic we also provide care for a 16-year-old female with DLD deficiency who developed severe post-crisis dystonia. This patient has the same compound heterozygous finding. It is our experience that the c.158G > A (p.Gly53Glu) variant, found among Libyan Jews, is a negative modifier of the common Jewish-Ashkenazi variant, c.685G > T (p.Gly229Cys). The c.685G > T (p.Gly229Cys) variant is usually associated with a mild course of disease, characterized by a later onset of metabolic crises, and a complete recovery in between crises without neurological sequelae [24].

*Cell phenotype and mitochondrial function characterization.*

To phenotypically characterize the skin fibroblasts sample from the male DLDD patient we applied image-based High Content analysis (HCA) in a similar way as previously described by us [25,26].

**Fig. 1:**
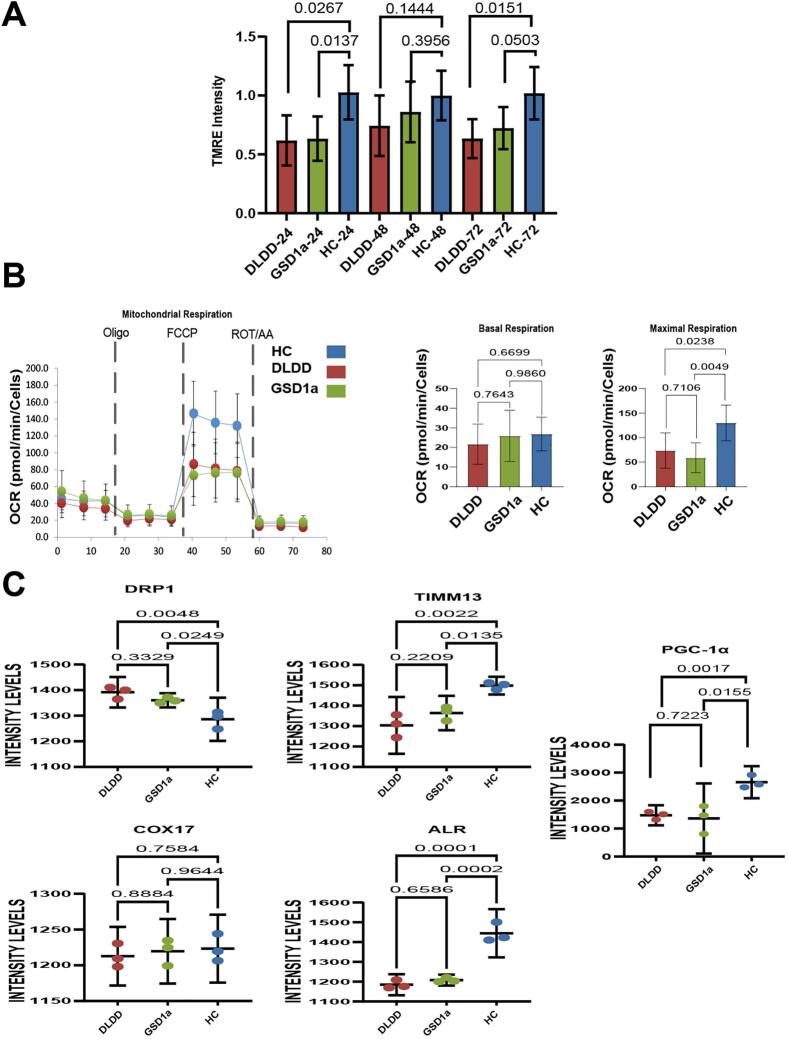
**A.** Bar plots with 95 % confidence intervals of TMRE intensity comparing DLDD, HC and GSD1a samples from all tested conditions. (N represents number of wells analyzed; 24 h-DLDD = 18, GSD1a = 32, HC = 38. 48 h- DLDD = 17, GSD1a = 29, HC = 36. 72 h- DLDD = 24, GSD1a = 33, HC = 36). **B.** Left- Representative quantification of Seahorse real-time cellular oxygen consumption rates (OCR) of GSD1a (green), DLDD (red) and HC (blue) fibroblasts. Dashed lines indicate the addition of mitochondrial inhibitors (oligomycin; FCCP; antimycin A/rotenone) for the respective assessments of ATP generating, total and non-mitochondrial respirations. Right- Bar plots of OCR-derived features with 95 % confidence intervals identified from GSD1a, DLDD and HC samples. (From left to right- Basal respiration, Maximal respiration). (N represents wells analyzed; DLDD = 6, GSD1a = 6, HC = 6). **C.** Scatter plots with 95 % confidence intervals of DLDD, GSD1a and HC cell lines comparing the levels of mitochondrial markers. Shown are DRP1, TIMM13, COX17, ALR and PGC-1α intensity levels as measured by immunofluorescence. (N represents wells analyzed; DLDD = 3, GSD1a = 3, HC = 3).

For this purpose, we analyzed skin fibroblasts samples from the DLDD patient, two GSD1a patients and three healthy controls (HC) (age and gender matched, see Table 1). The GSD1a patients' fibroblasts were used as a reference disease control as we recently established that these cells show a strong mitochondrial phenotype by HCA, The cell samples were incubated for 24 h, 48 h and 72 h following starvation protocols as detailed in Methods. These different culture conditions were used to induce metabolic stress in the cells caused by both sugar deprivation (24 and 48 h) and by glycogen burden caused by the addition of sugar to the media for 24 h after 48 h starvation (72 h), which proved to be highly effective for characterizing the GSD phenotype by HCA as in previous studies [21,22]. Plated cells were simultaneously co-stained with live fluorescent dye mix to label nuclei (Hoechst 33342 blue), cytoplasm (Calcein-AM green) and active mitochondria (TMRE red) and then multiple images for each well were taken to characterize organellar features for each culture condition. Representative images and data analysis of the different cellular imaging features from these experiments are shown in Supplementary Fig. 1. To focus on mitochondrial activity, which is most relevant for the DLDD cellular phenotype, we compared TMRE intensity levels between DLDD, GSD1a and HC fibroblasts for the three different culture conditions as shown in Fig. 1A. A clear reduction in TMRE intensity was observed in the DLDD patient fibroblasts in all tested conditions as compared to HC. At the 48 h condition possibly due to the largest starvation stress in all conditions, differences between DLDD and HC fibroblasts were the smallest (*p* < 0.144), possibly due to the relatively high starvation stress overwhelming inter-group differences. Interestingly, similar to DLDD, GSD1a fibroblasts also show low TMRE intensity levels as compared to HC, highlighting the similarity in lower mitochondrial activity in both metabolic disorders reflecting their respective cellular pathologies.

To further explore the mitochondrial activity in these cells, we performed Seahorse extracellular flux experiments in a similar manner as previously done by us [21,22].

We measured oxygen consumption rate (OCR) at the 72 h culture condition as shown in Fig. 1B. Mitochondrial activity in the DLDD line 53,273, and the GSD1a line 75,858, as compared to the HC line 498 (Table 1), is compromised as shown by the reduced overall oxygen consumption rate (Fig. 1B left). While basal respiration was not significantly different between the groups, the maximal respiration rate was significantly lower in the DLDD and GSD1a lines, as compared with the HC line (Fig. 1B right). To further investigate this phenotype, we tested in these fibroblast cell lines several markers related to mitochondrial physiology in these cells by immunofluorescence HCA using specific antibodies against DRP1, a mitochondrial fission marker [27], ALR (GFER), a marker of enzyme folding in the mitochondrial disulphide relay system [28], the respective mitochondrial protein import substrates TIMM13 and COX17 and the transcription factor which induces mitochondrial biogenesis, PGC1-α (Fig. 1C and representative images in Supplementary Figs. 2,3 and 4). Most of these markers show differences in levels in the DLDD patient as compared to HC. The increased DRP1 levels in DLDD may indicate higher mitochondrial fission, while the reduced ALR/GFER levels may cause the observed reduction in the levels of TIMM13, normally correctly folded by the ALR1 mitochondrial disulphide relay system. The levels of COX17, on the other hand, are also reduced, but not at a statistically significant levels, possibly also related to the ALR1 deficiency observed in DLDD fibroblasts. The observed reduction in PGC1-α, probably orchestrates this mitochondrial deficiency in DLDD fibroblasts at the transcriptional level because COX17 and other essential mitochondrial proteins depend on downstream concerted co-expression of other facilitators for their mitochondrial import and correct folding pathway.

*Differential transcriptomics analysis.*

**Fig. 2:**
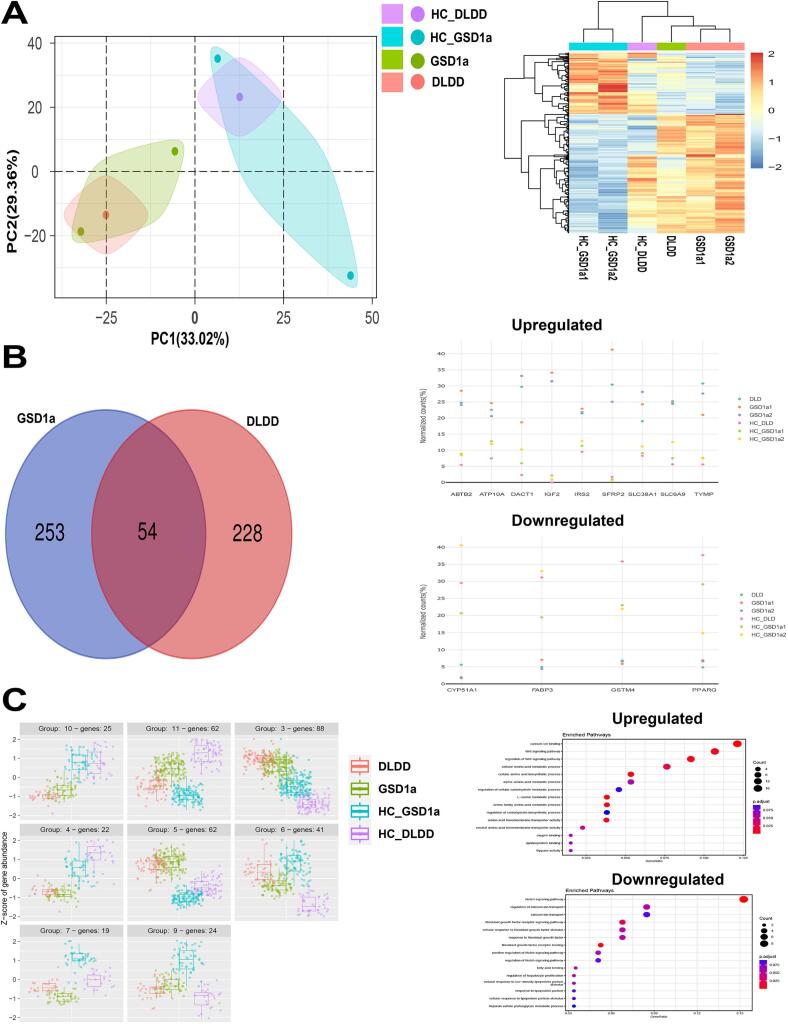
**A.** Left - PCA plot of RNA-seq data of DLDD (red), GSD1a (green) and healthy control (purple- DLDD control and pale blue- GSD1a controls) cell lines. Right- cluster heatmap of DLDD (red), GSD1a (green) and healthy control (purple- DLDD control and cyan- GSD1a controls) cell lines from the RNA-seq experiment. Dendrograms on the left side of the heatmap showcase cell line and feature similarity. **B.** Left - Venn diagrams of significant differentially expressed genes (FDR < 0.1) in DLDD and GSD1a. Right- scatter plots of significantly differentially expressed genes (FDR < 0.1) from the overlapping 54 genes shown in the Venn diagram. Gene values are presented as a percentage from total. The upper panel represents up-regulated genes and the lower panel represents down-regulated genes. **C.** Left - Box plots from a Likelihood ratio test (LRT) comparing DLDD (red), GSD1a (green) and healthy control (purple- DLDD control and cyan- GSD1a controls) cell lines. Y axis represents z scores from gene counts. Clusters represent significant differentially expressed genes (FDR < 0.1) from the LRT analysis, with different patterns between groups. Right - Dot plots from an enrichment analysis performed on genes from the LRT analysis. Upregulated clusters (upper panel- cluster 3 and 5) and downregulated clusters (lower panel- cluster 10, 7, 4) were compared to the GO database (*p* value was adjusted using FDR).

Next, we performed an RNA-seq experiment on one DLDD, two GSD1a, and three age and gender matching HC fibroblasts lines (Table 1) as shown in Fig. 2. Fig. 2A left shows a principal component analysis (PCA) performed on the full set of genes identified from the RNA-seq experiment. This analysis presented clear separation between the HC groups and the disease groups in PC1 with 33 % variance explained. In addition, the analysis showed larger variation in the HC group as compared to the disease fibroblasts indicating that fibroblasts from the DLDD and GSD1a patients present a more uniform inter-patient disease phenotype. Hierarchical clustering analysis (Fig. 2A right) was performed to assess this similarity between DLDD and GSD1a fibroblasts. This analysis revealed clusters of genes which are unique to each disorder and confirmed that the disease cells are more similar to each other than each one is to the HC samples. To investigate specific genes which are up or down regulated in the diseases compared to HC, we implemented conventional differential expression analysis using DESEQ2 where we compared each disease group with their matching HC fibroblasts. The rationale behind this experimental design was to identify differentially expressed genes that are related to the disease and not to age or gender. Supplementary Fig. 5 shows volcano plots for this analysis where hundreds of genes were significantly different (FDR < 0.1) in each comparison between groups (see Supplementary Table 1 and 2 for full list of significant genes). We then used these genes to generate Venn diagrams as shown in Fig. 2B (left). This analysis presented 54 significantly expressed genes which were shared by both diseases compared to healthy controls (see Supplementary Table 3 for list of overlapping genes). Fig. 2B (right) shows scatter plots of top genes identified in this analysis. In the upper panel, up-regulated genes are shown that are related either to metabolic activity, such as – ABTB2, ATP10A, TYMP, SLC38A1, SLC6A9, or to the Wnt signaling pathway, such as DACT1 and SFRP2, and insulin related genes – IGF2 and IRS2. In the bottom panel, down-regulated genes are shown. Those genes are related to fatty acid metabolism- PPARG and FABP3, and to protein metabolism – CYP51A1 and GSTM4. To expand the analysis of differential gene expression, taking into consideration the statistical limitations due to the reduced sample numbers (1 DLDD and 2 GSD1a), we used known methods such as likelihood ratio test (LRT), and DEGreports (see Methods). Fig. 2C (left panel) shows clusters of genes with strong similarities between the tested groups indicated. See Supplementary Table 4 for list of genes from relevant clusters 3,4,5,7,10. Next, to link these genes to cellular pathways that might characterize DLDD and GSD1a we applied an enrichment analysis of up-regulated genes in DLDD and GSD1a compared to HC (Fig. 2C, left panel, clusters 3 and 5) and of down-regulated genes in DLDD and GSD1a compared to HC (Fig. 2C, left panel, clusters 10, 4 and 7). Results from this analysis (right panel of Fig. 2C) show enriched pathways of up or down-regulated genes as dot plots. Interestingly, the up-regulated gene clusters enrichment analysis showed several pathways related to amino acid metabolism and to metabolic pathways in general while the down-regulated pathways showed fatty acid metabolism pathways and pathways related to cellular growth and overall viability. These results suggest a strong disease phenotype at the transcription level indicating metabolic stress in both the DLDD and GSD1a samples.

*Metabolomics.*

**Fig. 3:**
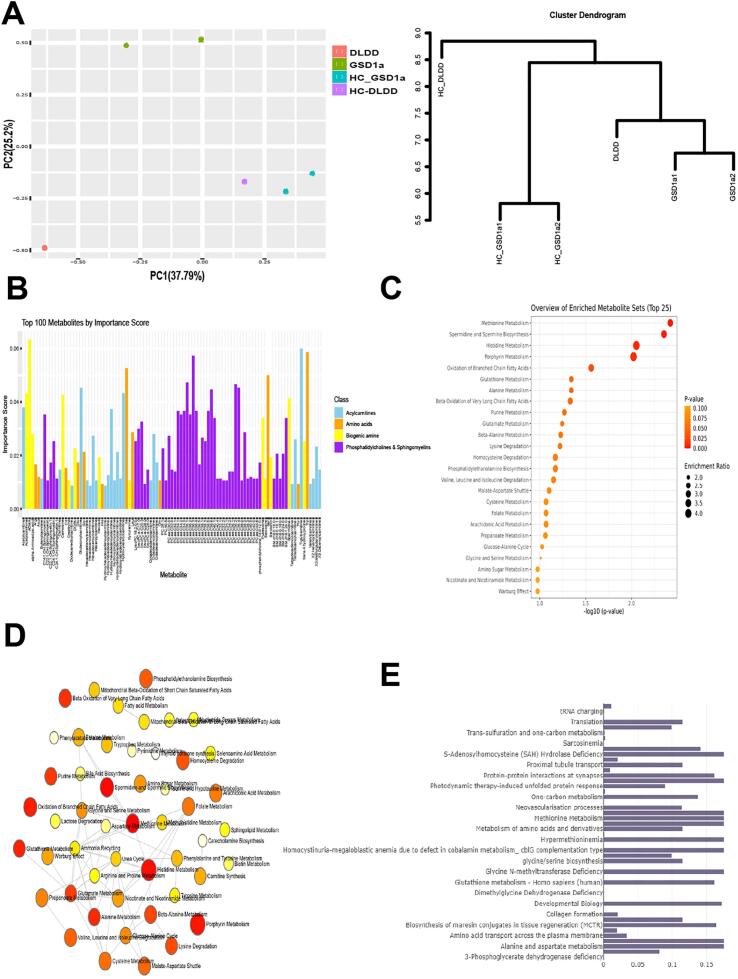
**A.** Left – PCA plot of metabolomics data. The PCA plot presents DLDD (red), GSD1a (green) and healthy control (purple- DLDD control and cyan- GSD1a controls) cell lines. Right- cluster dendrogram presenting DLDD, GSD1a and healthy control cell lines from the metabolomics experiment. **B.** Bar plots indicating importance scores of the top 100 metabolites from the metabolomics dataset as computed using Random Forest for classifying DLDD/GSD1a samples. Classes of metabolites are shown to the right of the plot. **C.** Metabolite Set Enrichment Analysis (MSEA) for extracts from fibroblasts of GSD1a/DLDD compared to HC fibroblasts. **D.** An interactome of enriched pathways predicted by MSEA. **E.** Bar plots of results from a pathway-integrated analysis of RNA-seq differentially expressed genes from DLDD patient, and DLDD differentiating metabolites from the metabolomics dataset.

To corroborate the results shown above and to explore the metabolic aberrations in DLDD/GSD1a fibroblasts, we profiled the metabolomic landscape of DLDD/GSD1a patients as compared to HC subjects (Fig. 3). Fig. 3A shows a PCA and a cluster dendrogram implemented on the full dataset of metabolites. Results from this analysis showed a strong separation between HC and patient samples, but also showed a unique phenotype for the DLDD sample as compared to either GSD1a cells or to HC (see Supplementary Table 5 for full data set of metabolites analyzed). Due to the paucity of DLDD clinical samples, and since a minimum of three samples per group can be analyzed by Metaboanalyst 6.0, we analyzed all patient samples together as a single group. Random Forest classification was performed on patient and HC groups generating scores for importance of metabolites for group classification. This enabled us to find metabolites which are mostly relevant for disease classification. The results of this analysis are presented in Fig. 3B as bar plots of the top scoring 100 metabolites of importance. These metabolites are plotted according to their respective metabolic class, class 1: acylcarnitines; class 2: amino acids; class3: biogenic amines; and class 4: phosphatidylcholines and sphingomyelins.

We used the Metaboanlyst 6.0 metabolite set enrichment analysis (MSEA) against the SMPDB 99 metabolite set to predict the major metabolic pathways modified by the diseased state (Fig. 3C) and the interactions among them (Fig. 3D). Two of these pathways are likely to play a major role in DLDD and GSD1a pathophysiology: *methionine metabolism* (https://www.smpdb.ca/view/SMP0000033, enrichment significance *p* < 0.003, FDR < 0.11), and *spermidine and spermine biosynthesis* (https://smpdb.ca/view/SMP0000445, enrichment significance *p* < 0.004, FDR < 0.11)*.* Inhibition of methionine metabolism was computationally deduced from the relative changes in several metabolites, such as methionine accumulation and glycine accumulation (Supplementary Fig. 6 A). Methionine metabolism is directly related to glycine cleavage because the latter activates one carbon (methyl) transfer to the folate cycle, which is coupled to the methionine cycle [29]. These two interlinked cycles comprise together the one‑carbon cycle [30]. A key metabolic feature of the methionine cycle is production of S-adenosylmethionine (SAM), which furnishes methyl for the biosynthesis of DNA, amino acids, phospholipids and creatine, as well as for DNA methylation, essential for epigenetic regulation [31]. Thus, deficient methionine and one‑carbon metabolism in DLDD probably has a major pathophysiological role. Inhibition of the spermidine and spermine biosynthesis pathway was deduced from changes in the polyamines putrescine and spermidine and the amino acids methionine and ornithine (Supplementary Fig. 6B). This pathway is also important because of the link to methionine metabolism and SAM production. The accumulation of methionine, suggesting its reduced turnover to SAM within the spermidine and spermine biosynthesis pathway, can possibly contribute to DLDD pathophysiology. Another important example is the reduced extent of branched amino acid degradation (https://www.smpdb.ca/view/SMP0000032), as revealed by the accumulation of Leu and Ile and the decrease in glutamic acid catabolized, or degraded, from Valine (Supplementary Fig. 6C). Degradation, or metabolization, of the branched amino acid Leu to acetylCoA can bypass PDH production of acetylCoA. Thus, Leu degradation serves as an important metabolic compensation mechanism in times of fuel stress, or deficiency in OxPhos energy substrates. As the difference in the levels of glutamic levels are less pronounced in DLDD cells, inhibition of branched chain amino acid degradation is probably less evident in DLDD as compared to GSD1a. The interactome presented (Fig. 3D) is very informative as it can potentially explain connectivity and negative and positive feedbacks between pathways which share common metabolites. As an example, related to the one‑carbon metabolism, Methionine metabolism is interlinked with Spermidine and spermine biosynthesis, but between them only methionine metabolism is interconnected with folate metabolism (https://smpdb.ca/pathwhiz/pathways/PW000024), which has no metabolite in common with the Spermidine and spermine pathway in the same compartment (tetrahydrofolic acid is formed in the mitochondria within the Methionine metabolism pathway and in the cytosol within the Folate metabolism pathway). Interestingly, as can be observed in Fig. 3D, the Valine, Leucine and Isoleucine (branched amino acids) degradation pathway is not interconnected with the key metabolic aberrations observed in GSD1a and DLDD (one‑carbon related pathways), suggesting that it might stand alone also as a therapeutic target unrelated to one‑carbon metabolism.

To explore the relevance of both the metabolite data set and the RNA-seq data set for the DLDD cell sample, we implemented an integrated pathway analysis using IMPALA combining differential genes from both GSD1a and DLDD samples with metabolites with differences between both disease groups. Results from this analysis are shown as a bar plot in Fig. 3E. Among the pathways identified by this analysis are metabolic pathways related to broad amino acid metabolism and cellular processes such as translation, which depends on the Methionine metabolism and Folate metabolism pathways for the respective provision of Methionyl-tRNA and 10-Formyltetrahydrofolate for N-formylmethionyl-tRNA (fMet-tRNA). Thus, deficiencies in these metabolic pathways are in line with the known cellular effects of metabolic disorders.

## 4 Discussion

Our meta-analysis of primary skin fibroblasts from the DLDD patient shows a multilayer description of the DLDD cell phenotype. The first layer shows image based-HCA phenotypic characterization of active mitochondrial features that were lower in DLDD and GSD1a patients' fibroblasts as compared to HC fibroblasts, indicating, as expected, a mitochondrial disease related phenotype for both DLDD and GSD1a metabolic disorders. This observation was confirmed by the Seahorse analysis that directly measured OCR in these cells showing that the maximal respiration rate was significantly lower in DLDD and GSD1a fibroblasts, as compared with HC fibroblasts. This result might indicate that DLDD fibroblasts are under stress at their maximal energy output even after 24 h restoration of high glucose medium conditions following 48 h starvation. The observed reduction in mitochondrial function is expected from a disorder affecting pyruvate dehydrogenase (PDH) and α-ketogluterate dehydrogenase (α-KGDH) [32]. These two enzymes link glycolysis to oxidative phosphorylation (OxPhos) by turning glycolysis derived pyruvic acid into acetyl CoA, and facilitating flux through the Krebs cycle, respectively. OxPhos is significantly affected in DLDD patient's fibroblasts as shown by the respiratory effects on the mitochondria and by the attenuated glycolytic acidification [1,31,33]. Importantly, DLD is the E3 subunit of PDH. Therefore, DLDD must have profound metabolic consequences because NADH and most acetyl-CoA in the cell are generated in the reaction catalyzed by PDH. In addition, DLD is also a dehydrogenase of alpha-ketoglutarate, one of the rate limiting enzymes of the Krebs cycle flux. In other words, DLD controls the Krebs cycle which might explain the changes in pathways generally associated with mitochondrial respiratory dysfunction, such as Oxidation of Branched Chain Fatty Acids (Fig. 3C), which is not connected to deficiencies in the one‑carbon pathway, such as Methionine, or Folate metabolism (Fig. 3D). Moreover, this metabolic stress status might be supported by abnormal mitochondrial protein activities in DLDD and GSD1a involving high DRP1 that may directly influence mitochondrial fusion-fission dynamic balance, resulting in more fragmented non-viable mitochondria, obliterating mitochondrial recycling and biogenesis in DLDD cells. Low ALR levels may have an impact on TIMM13 and COX17 levels that are known substrates of the ALR mediated MDRS folding machinery of imported proteins in the mitochondria. Interestingly, these results support global mitochondrial dysfunction in the patient's fibroblasts, correlating to findings in patients with pyruvate oxidation defects [34]. Transcriptome analysis of patient and HC cells importantly showed that the two disorders DLDD and GSD1a are more similar to each other than each one is to the HC samples. Conventional differential expression analysis, comparing each disease group to their matching HC cells showed significant differences between groups which presented at least 54 significantly expressed genes (from thousands expressed) which were shared by both diseases compared to HC. The up-regulated genes were found to be related to inhibitors of the Wnt signaling pathway, to response to insulin signal, and to various metabolic activities such as the expression of cation amino acid transporters and Thymidine phosphorylase (TYMP), mutations in which are associated with mitochondrial neurogastrointestinal encephalomyopathy (MNGIE) [35]. The down-regulated genes were found to be related to fatty acid and protein metabolism. Additionally, alterations in lipid metabolism and specific amino acid catabolism (*e.g.*, glycine and serine) are commonly observed in mitochondrial dysfunctions [36]. Having in mind the statistical limitations due to the reduced number of available samples (from a single DLDD and 2 GSD1a patients), we performed an adapted testing for differential gene expression between all groups. Using these tests and clustering methods, we identified clusters of genes linked to cellular pathways that might phenotypically characterize DLDD and GSD1a when they are up-regulated, or down-regulated compared to the GO database. Using these analyses, we discovered that the up-regulated gene pathways were related to amino acid metabolism while the down regulated pathways were related to fatty acid metabolism, cellular growth, and overall viability. These results suggest a strong disease phenotype at the transcription level, indicating metabolic stress in both the DLDD and GSD1a samples. The metabolomics results for DLDD and GSD1A samples corroborated our conclusion on the metabolic stress state of patient cells by showing several key metabolites classes that were affected in these disorders in skin fibroblasts. The integrated pathway analysis shown here for the metabolite data set and the RNA-seq data set for the DLDD cell sample identified metabolic pathways, identified by MSEA against the SMPDB dataset (see Methods), related to broad amino acid metabolism and cellular processes such as translation, which are known cellular effects in metabolic disorders. Interestingly, the up-regulation of amino acid metabolism, especially serine and serine/threonine ratio found in our amino acid metabolite class, might relate to the modified expression of 3-phosphoglycerate dehydrogenase, which off shoots from the glycolytic pathway and uses the glycolytic substrate 3-phosphoglycerate to generate serine. Cluster metabolomic analysis, including the finding of 3-phosphoglycerate dehydrogenase deficiency, adds another layer in mitochondrial dysfunction in DLDD fibroblasts. Together with this, we found Glycine cleavage dysfunction. This process requires DLD as part of the 4 enzymes implicated in it. Here we show the accumulation of glycine metabolites in DLDD cells. These results may shed light on the importance of this pathway in the disease pathophysiology, which so far hasn't been studied in DLDD, and thus shows a promising clinical interest. In addition to the already established glycine cleavage deficiency in DLDD, we present here a yet unknown biochemical aspect of DLDD, related to one‑carbon metabolism, involving changes in methionine, SAH hydrolase, glutathione findings and up-regulation of tRNA synthesis, which might compensate for the amino acid imbalance in the cells as described above. We do not know the clinical significance of these findings. However, we believe that this information might be important to follow in further studies of patients with DLDD. It is possible that these differential pathways are activated in response to mitochondrial dysfunction caused by metabolic stress which serves as a reprogramming signal triggering the observed compensatory response in DLDD fibroblasts.

Importantly, this study was conducted in skin fibroblasts, which are an accessible tissue expressing the mutant DLD. As DLDD is not a skin disorder, our findings may not recapitulate the etiology of the disease in pathophysiological relevant tissues such as liver or muscle. Thus, while the metabolic and organellar phenotyping of DLDD fibroblasts reflects the findings shown in the DLDD patient fibroblasts, their therapeutic implementation may require further experiments, for instance in inducible tissue-targeted DLDD mouse models.

In conclusion, the novel personalized multi-omics analysis described in this study, utilizing primary fibroblasts from a single DLDD patient and GSD1a patients (as valuable multi-omics disease reference controls), provides important biological disease-related data that could enhance the clinical assessment of patients with rare diseases. This approach adds a new dimension of cell-based personalized information for rare genetic disorders like DLDD, potentially improving individualized clinical evaluation for patients with such rare conditions.

## CRediT authorship contribution statement

**Uri Sprecher:** Writing – review & editing, Writing – original draft, Visualization, Software, Methodology, Investigation, Formal analysis, Data curation, Conceptualization. **Jeevitha Dsouza:** Methodology, Investigation, Data curation, Conceptualization. **Monzer Marisat:** Investigation, Data curation. **Dinorah Barasch:** Methodology, Formal analysis, Data curation. **Kumudesh Mishra:** Methodology, Investigation, Data curation. **Or Kakhlon:** Writing – review & editing, Validation, Supervision, Methodology, Funding acquisition, Conceptualization. **Joshua Manor:** Writing – review & editing, Writing – original draft, Validation, Supervision, Data curation, Conceptualization. **Yair Anikster:** Writing – review & editing, Writing – original draft, Supervision, Project administration, Funding acquisition, Data curation, Conceptualization. **Miguel Weil:** Writing – review & editing, Writing – original draft, Validation, Supervision, Project administration, Funding acquisition, Conceptualization.

## Declaration of competing interest

The authors declare that they have no known competing financial interests or personal relationships that could have appeared to influence the work reported in this paper.

## Data Availability

Data will be made available on request.
