# Supplementary figures and images for: In depth profiling of dihydrolipoamide dehydrogenase deficiency in primary patients fibroblasts reveals metabolic reprogramming secondary to mitochondrial dysfunction
Source: Mol Genet Metab Rep. 2024 Dec 16;42:101172. doi: 10.1016/j.ymgmr.2024.101172 (PMC11719413; doi:10.1016/j.ymgmr.2024.101172)

# B

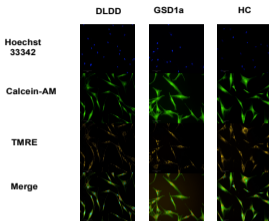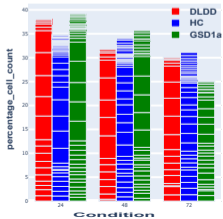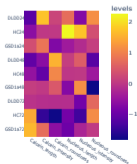

Supplement: Supplementary material 1 — Supplementary Fig. 1 [file mmc1.pdf]

Hoescht3342 Phalloidin COX17 Merge

HC

DLDD

GSD1a

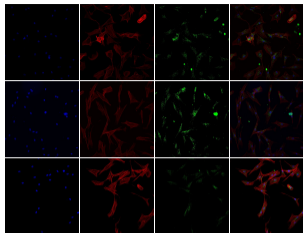

Hoescht3342 Phalloidin TIMM13 Merge

HC

DLDD

GSD1a

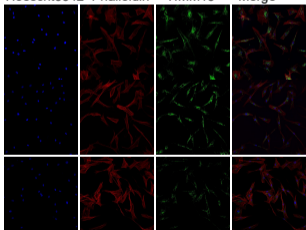

Supplement: Supplementary material 2 — Supplementary Fig. 2 [file mmc2.pdf]

Hoescht3342 Phalloidin DRP1 Merge

HC

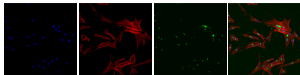

DLDD

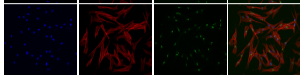

GSD1a

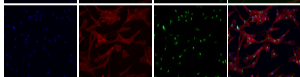

Hoescht3342 Phalloidin ALR Merge

HC

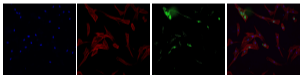

DLDD

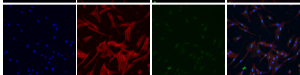

GSD1a

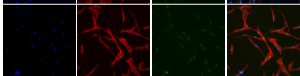

Supplement: Supplementary material 3 — Supplementary Fig. 3 [file mmc3.pdf]

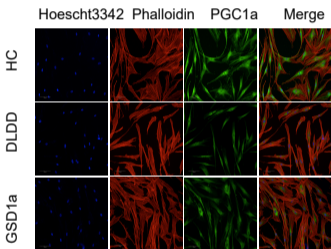

Supplement: Supplementary material 4 — Supplementary Fig. 4 [file mmc4.pdf]

## HC vs DLDD

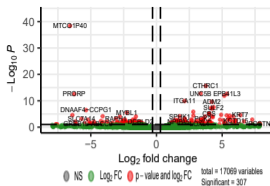

## HC vs GSD1a

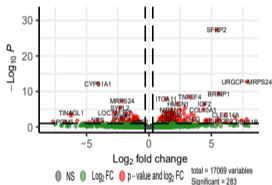

## GSD1a vs DLDD

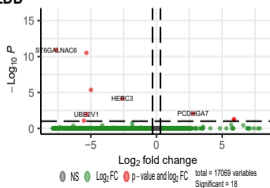

Supplement: Supplementary material 5 — Supplementary Fig. 5 [file mmc5.pdf]

**A**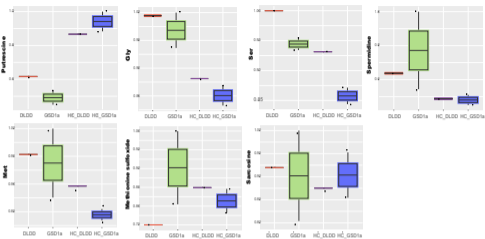**B**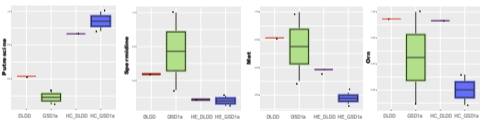**C**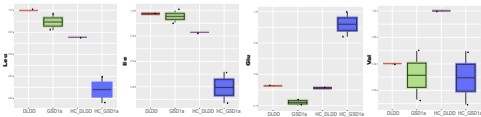

Supplement: Supplementary material 6 — Supplementary Fig. 6 [file mmc6.pdf]
